# Supplementary material for: Are carbon emissions trading and green financial instruments synergistic? -Comprehensive quantitative research based on content analysis
Source: PLoS One. 2024 Mar 7;19(3):e0298601. doi: 10.1371/journal.pone.0298601 (PMC10919601; doi:10.1371/journal.pone.0298601)
Supplement: S1 Data — (ZIP) [file pone.0298601.s001.zip › Data/地级市Z_F工作报告.zip!/║╙─╧/╔╠╟≡╩╨/└┤╫╘╣1⁄2╓┌║┼í╛┬φ┐╦╩2╛▌═°í┐_2011.docx]

各位代表：

现在，我代表市人民政府，向大会报告工作，请予审议，并请各位政协委员和其他列席会议的同志提出意见。

一、2010年及“十一五”工作回顾

2010年是我市经济社会发展不寻常的一年，面对复杂形势和诸多困难，在省委、省政府和市委的正确领导下，全市上下坚持以扩内需、调结构、促转型为主线，以产业集聚区、招商引资、重点项目、城镇建设四项工作为重点，抢抓机遇，扎实工作，较好地完成了市三届人大五次会议确定的各项目标任务，经济社会保持了好的趋势、好的态势、好的气势。初步预计，全市生产总值完成1140亿元，比上年增长12％左右；全市财政总收入83．7亿元，增长18．8％，财政一般预算收入43亿元，增长21．6％，一般预算支出180亿元，增长19．1％。城镇居民人均可支配收入增长11．5％，农民人均纯收入增长15．3％。

（一）经济持续平稳较快发展。面对复杂多变的经济环境，我们采取了针对性较强的措施积极应对，年初以保障电煤供应为重点，全力支持企业开足马力生产，一季度经济运行实现了开门红，生产总值同比增长16．1％；5月份以后，随着货币政策和行业优惠电价政策的调整，工业经济发展遇到了困难，我们把遏制工业经济增速回落作为经济运行的关键环节，积极帮助骨干企业争取大用户直供用电，加强银企合作，服务企业开拓市场，全年完成新增贷款88．72亿元、新增资本市场融资51亿元；10月份以来，在电力供应日趋紧张的压力下，坚持区别对待，有保有压，保证了居民、农业和重点领域的电力供应，企业正常生产经营和居民生活没有受到影响。去年全市规模以上工业增加值完成359．6亿元，增长17．9％；克服低温多雨等不利天气影响，粮食生产再创历史新高，总产达到123亿斤；城镇固定资产投资690．39亿元，增长23．1％。

（二）四项重点工作成效明显。按照招商引资有大活动、产业集聚区有大变化、项目建设有大突破、城镇建设有大动作的要求，扎实推进四项重点工作。招商引资成果丰硕，组织参加了豫浙经贸洽谈会、深港澳闽籍企业家访豫、河南台湾月、中国郑州2010产业转移系列对接等活动，围绕出新、出彩、出效，成功举办2010中国�商丘国际华商节。全年招商落地5000万元以上项目232个，到位省外资金200．9亿元，总投资12亿元的香雪海年产300万台冰箱冷柜项目实现了百日竣工投产，完成了香雪海生产基地向我市的整体搬迁。产业集聚区建设速度加快，大力推进基础设施建设，完成基础设施投资50．55亿元，使每个产业集聚区原计划2亿元的基础设施年度投资目标翻了一番。全市11个产业集聚区新入驻企业246家，营业收入实现650亿元以上。在全省产业集聚区观摩评比活动中我市取得好成绩，柘城县、民权县产业集聚区跨入全省示范产业集聚区行列。重点项目建设进展顺利，深入开展“项目提升年”活动，254个省、市重点项目累计完成投资337亿元。商周高速二期工程等129个项目开工建设，庄周50万伏输变电工程二期等132个项目建成投产，中石油商丘千万吨炼油项目列入河南省和中石油集团战略合作协议，正在努力纳入国家“十二五”规划；商丘机场项目上报国务院、中央军委待批；千吨级碳纤维项目8个月建成投产，创造了商丘速度。城镇面貌发生较大变化，围绕做靓中心城区、做美县域城区、做特中心镇区、做新农村社区的目标，加快推进城镇建设，中心城区功能不断完善，城镇框架进一步拉大，一些县城及中心乡镇面貌发生了日新月异的变化。全市城镇化率达到37．8％，比上年提高2．3个百分点，城镇吸纳和辐射能力不断增强。

（三）“三农”工作取得新的成绩。坚持把“三农”工作放在重要位置，努力实现粮食增产、农业增效、农民增收。粮食综合生产能力得到提高，实施粮食生产发展规划，抓好粮食生产核心区建设，扩大优质小麦种植面积，粮食综合生产能力稳定在120亿斤左右，完成粮食收购40．2亿斤，带动农民增收1．6亿元。农业产业结构得到不断优化，大力推进农业标准化种植和规模化养殖，增加果蔬、油料、花卉等经济作物面积，畜牧业产值占农业总产值的比重提高到30％以上。全市林木覆盖率达到28．6％，在全省率先建成林业生态市。农业产业化步伐逐步加快，重点扶持30家农副产品加工龙头企业，培育了一批年产值超亿元的企业，全市农业产业化重点龙头企业达到237家，省级以上达到49家，农民专业合作组织达到1586家，32家被评为全省首批农民专业合作社示范社。农业基础设施建设进一步加强，投入水利建设资金7．2亿元，实施了吴屯、石庄水库除险加固和三义寨引黄灌区节水改造等工程，第16次荣获省“红旗渠精神杯”。新建农村户用沼气1万多座。解决了农村54．4万人的饮水安全问题。全市农机总动力达到1123．2万千瓦，居全省第二位。改造中低产田8万亩，建设高标准农田4万亩，农业抗御自然灾害的能力进一步增强。新型农村社区建设稳步推进，完成4626个行政村的村庄发展建设规划，组织开展了以道路、电网、通信网络为重点的农村基础设施升级改造。实施“扶弱培强”工程，抓好“百村示范”建设，开展“清洁家园”行动，农民生产生活条件得到改善。

（四）落实政策措施及时有效。认真落实国家围绕保增长、调结构、减排放、稳物价出台的一系列调控政策。抓好节能减排，突出重点领域、重点行业和重点企业，健全资金投入、政策引导、监督管理、目标考评机制，加强生态环保基础设施和节能重点工程建设，预计万元GDP能耗降低到1．14吨标准煤，比上年下降4．3％。大气质量得到进一步改善，城区空气质量优良率为90．4％。COD和SO2减排目标圆满完成。推进转型升级，加快推进主导产业发展，增强企业自主创新能力，带动全市工业结构优化升级；依托优势产业，加强与央企及国内外优势企业的战略合作，促进市内骨干企业联合重组，培育壮大行业地位突出的大型企业集团；加快碳纤维、电动汽车、新型电池、生物能源、生物制药等新兴产业的发展，培育新的经济增长点。实施质量兴市战略，开展首届市长质量奖评比和“千企百家质量行”活动，全市名牌产品达到30个，省级著名商标达到66个。加快发展现代服务业，加强文化产业园区和示范基地建设，促进旅游产业大发展，黄河故道湿地水利风景区被批准为国家级水利风景区。加强物价监控，以落实调控政策、加强行业监管、维护市场稳定为重点，科学引导房地产投资和消费，全市房地产开发投资78．4亿元，增长31．4％，销售价格增长平稳。积极抓好基本生产生活必需品的保障供应，落实好鲜活农产品“绿色通道”及收费减免政策，维持了全市整体价格水平，生活必需品价格保持平稳。

（五）改革开放进程不断深化。注重改革创新体制机制，深入实施开放带动主战略。突出重点领域改革，市、县政府机构改革圆满结束，公安警务机制改革取得明显成效。深化行政审批制度改革，把市级保留的行政审批事项、行政事业性收费事项全部纳入电子监察系统管理，规范了审批行为，提高了审批效率。积极推进国有企业改革和战略重组，壮大主业规模，实现优势互补。文化体制改革继续推进，医药卫生体制改革完成阶段性目标。加快对外开放步伐，发挥区位优势，加强与淮海经济区、陇海兰新经济促进会、大京九经济协作带的战略合作，加强与央企及国内大企业、大集团的合作，飞龙电器、沃尔玛、红星美凯龙等一大批知名企业落户商丘。我市被省政府评为对外开放优秀单位、招商引资工作先进单位。优化经济发展环境，制定完善招商引资政策，加大案件查办和企业周边环境综合整治力度，及时解决影响经济发展的环境问题。通过民意快线、市长热线、市长信箱和政风行风热线畅通民意诉求渠道，办结率、反馈率、满意率不断提高，区域经济发展环境综合考评工作在全省名列前茅。

（六）人民群众得到更多实惠。把发展经济与改善民生相结合，持续加大投入，集中财力办好省定十项民生工程和市定十件实事，兑现各项惠农补贴资金15．6亿元。优先发展教育事业，制定了教育、人才改革发展规划，义务教育人口覆盖率达到99．5％；高等院校达到6所，居全省第四位，华豫学院转设为独立设置民办本科院校，商丘科技职业学院专升本工作顺利通过教育部高评委评审，商丘职业技术学院通过全国示范性职业院校验收，我市被国务院确定为地方政府促进高等职业教育发展综合改革试点。实施积极的就业政策，全市城镇新增就业8．6万人，下岗失业再就业3．3万人，城镇登记失业率为3．58％；劳务输出220万人，创劳务收入140亿元。繁荣发展文化广电新闻出版事业和文化产业，文化服务体系逐步完善，新建乡镇文化站90个，文化信息资源共享工程扎实推进，广播电视有效覆盖面进一步扩大，代表河南省参加赴台文化交流圆满成功。进一步健全公共卫生服务体系，城乡卫生基础设施不断加强，新增中央投资县级医院、乡镇卫生院、社区卫生服务中心、村卫生室和精神卫生项目158个。扎实开展全民健身运动，积极参加省第十一届运动会，取得较好成绩。完善社会保障体系，社保覆盖面和最高支付限额大幅提高。新农合筹资水平提高到人均150元，参合率达到92％，睢阳区被纳入国家第二批新型农村养老保险试点。加快推进保障性住房建设，建成廉租住房8760套、经济适用住房5010套，完成棚户区改造25．26万平方米，灾后倒房重建任务如期完成。加强人口和计生工作，继续稳定低生育水平，出生人口性别比偏高问题得到有效遏制，人口自然增长率控制在4．77％。，全国计划生育优质服务先进县达到4个。

（七）社会大局保持和谐稳定。全面落实安全生产责任制，深入开展重点行业领域的安全整治，健全安全生产监管体制，严厉打击非法违法生产行为，全市安全形势平稳。加强食品药品安全管理，产品质量和食品药品安全保障水平继续提高。健全大调解机制，认真开展大接访、大排查、大化解活动，疏通群众上访渠道，在推进“事要解决”上下工夫，全市新发信访案件总量持续下降，重大敏感时期、重大活动期间没有发生来自商丘的干扰。以平安商丘建设为抓手，加强社会治安综合治理，严厉打击各类违法犯罪行为，社会治安环境明显改善。不断创新完善民族宗教工作机制，巩固了民族团结、宗教和睦、社会和谐的大局。

（八）民主法制建设和精神文明建设稳步推进。大力推进依法行政和政务公开，基层民主政治建设健康发展。自觉接受人大及其常委会的监督，支持政协履行职能，办理人大代表建议151件、政协委员提案631件，办结率100％。“五五普法”活动深入开展，依法治市全面推进。爱国统一战线得到巩固，各民主党派、工商联和工、青、妇等人民团体围绕大局，发挥作用，形成了团结奋进的良好局面。加强廉政建设，反腐倡廉工作取得新成效。广泛开展群众性精神文明创建活动，公民道德水平和文明程度不断提升。

大力开展国防教育，深入做好双拥工作。驻商解放军和武警部队在支持地方建设、维护社会稳定方面作出了重要贡献。外事、侨务、对台工作进一步加强，审计、供销、邮政、盐业、烟草、史志、地震、人民防空、档案管理、社科研究、青少年、妇女儿童、老龄、残疾人和慈善事业等工作都取得了新的成绩。第六次人口普查工作进展顺利。

各位代表，2010年各项任务的圆满完成，标志着“十一五”规划确定的主要目标胜利实现。“十一五”这五年，我们积极应对复杂局面，有效化解金融危机，战胜罕见自然灾害，较好地完成了“十一五”规划确定的主要目标任务，全市经济社会进入蓄势发展的崭新阶段。

——“十一五”期间，经济总量实现历史性突破。全市生产总值翻了一番，提前一年突破千亿元大关，年均增长12％，人均生产总值由7951元达到13800元；规模以上工业企业由2005年的334家增加到目前的810家，规模以上工业增加值由110．2亿元增长到359．6亿元，工业总产值超亿元的企业由68家增长到224家；财政一般预算收入由16．6亿元增加到43亿元，年均增长21％。

——“十一五”期间，结构优化实现历史性突破。在稳定农业的前提下，坚持走新型工业化、新型城镇化道路，以构建“一个载体、三个体系”为重点，深入推进经济结构调整和发展方式转变，产业结构不断优化，三次产业结构的比例由二一三调整为二三一。

——“十一五”期间，粮食产量实现历史性突破。加强粮食生产核心区建设，粮食产量连续5年稳定在百亿斤以上，占全省粮食总产量的1／9，居全省第三位，综合生产能力达到120亿斤，成为全国18个粮食总产量超百亿斤的地级市，国家粮食生产核心区地位进一步确立。

——“十一五”期间，城镇化进程实现历史性突破。加快城市基础设施和公共服务设施建设，全市城镇建成区面积达到322平方公里，城镇总人口达到313万人，全市城镇化率5年提高11．2个百分点，商丘正在向特大城市行列迈进。我市获得“中国优秀旅游城市”、“国家园林城市”、“全国绿化模范城市”、“全国双拥模范城”、“全国科技进步先进市”、“全国粮食生产先进市”、“全国民族团结进步先进集体”等荣誉称号，“华商之都”城市品牌的影响力和美誉度不断增强！

“十一五”时期取得的辉煌成绩，书写了我市全面建设小康社会的崭新篇章，奠定了商丘未来发展的良好基础，坚定了我们战胜困难的雄心壮志。这些成绩的取得，是省委、省政府和市委正确领导的结果，是人大、政协大力支持的结果，是全市上下齐心协力、共同奋斗的结果。在此，我代表市人民政府，向工作在全市各条战线上的工人、农民、知识分子、广大干部、人民解放军指战员、武警官兵、公安民警以及各界人士致以崇高的敬意！向参与商丘建设的各位投资者和所有关心支持商丘发展的海内外朋友，表示衷心的感谢！

各位代表，回顾“十一五”特别是去年的工作，我们经受住了国际金融危机影响和严重自然灾害考验，取得了来之不易的成绩，积累了弥足珍贵的经验。我们的体会主要有：一是咬定发展不放松。坚持把发展作为第一要务，作为解决前进中一切问题的关键，聚精会神搞建设，一心一意谋发展，努力在发展中调整结构，在发展中转型升级，在发展中提高质量，在发展中解决问题，千方百计保持经济又好又快发展。二是重点工作重点抓。注重以抓好重点工作带动全局发展，去年我市确定四项重点工作，实行了每月有通报、每季有观摩、半年有总结、年末有评比的工作制度，通过现场办公、观摩点评、互查互促的办法，抓两头、促中间，有针对性地解决工作中的问题，取得了比较好的成效。三是积极应对破难题。以特殊之举应对困难之局，打赢了战危机保增长的总体攻坚战；以体制机制改革和政策创新破解发展中的难题，努力解决资金、土地、人力保障方面存在的矛盾和问题；正确处理节能减排与调结构、促发展的关系，淘汰落后产能，增加优质产能，既最大限度保护了现实生产力，又维护了正常的生产生活秩序，防止了大起大落。四是备足后劲谋长远。坚持把保持经济平稳较快增长与实现长远发展目标结合起来，打牢基础，备足后劲，全市11个产业集聚区全部实现“三规合一”，重大项目不断增多，综合交通体系、中心城区生态水系规划建设工作正在加快推进。基础设施建设力度继续增强，投资体制改革深入开展，人才、信息等要素市场建设步伐加快，土地保障和服务发展能力得到进一步提升。五是改善民生办实事。把发展经济与改善民生结合起来，围绕解决人民群众关注的就业难、看病难、出行难等热点问题，尽心尽力为群众办实事、解难事，让人民群众得到更多的实惠，享受更多的发展成果，不断提高城乡居民的幸福指数。六是团结一致求实效。在实干中增进团结，在为民中凝聚人心，广大干部保持了合力攻坚、拼搏进取的良好状态，表现出了顾全大局、开拓进取的综合素质，呈现出团结一致、干事创业的发展局面，形成了脚踏实地、埋头苦干的区域优势。

各位代表，在肯定成绩的同时，我们也清醒地认识到，当前经济社会发展中还存在不少困难和问题：区域竞争压力越来越大，资源环境约束日益加剧，保持经济社会持续较快发展难度增加；节能降耗形势严峻，调整经济结构、转变经济发展方式任务繁重；社会事业和农村基础设施投入较少，社会保障能力和水平有待提高，经济社会发展的一条腿长、一条腿短问题需要下更大气力着力解决；政府工作还存在不少问题和不足，一些部门办事效率低，不作为、乱作为、慢作为，一些工作人员基本素质低，不得好处不办事，得了好处乱办事，群众反映办事难的问题依然突出。对此，我们一定高度重视，认真解决。

二、“十二五”发展总体思路和目标任务

市委三届十次全会通过的贯彻落实《中原经济区建设纲要（试行）》的实施意见和“十二五”规划建议，是指导商丘今后一个时期发展的行动纲领。在中原经济区建设中，我市的战略定位可以概括为“一个中心一个枢纽三个基地”，即建设豫鲁苏皖接合部区域性中心城市、中原经济区综合交通枢纽、国家粮食核心区重要生产基地、新兴工业基地和全国商贸物流集散基地。经济社会发展目标是“两高一低一提升”，即主要经济指标年均增速高于全省平均水平、高于豫鲁苏皖四省周边地区平均水平，人口自然增长率低于全省平均水平，提升在淮海经济协作区中的地位。战略步骤是五年初显优势，十年长足发展，构筑中原经济区东部战略支撑，成为又好又快发展的新的经济增长板块。

“十二五”时期是可以大有作为的重要战略机遇期，是加快经济发展方式转变的攻坚时期，也是中原经济区建设的奠基时期。这一阶段，我们既迎来产业转移、扩大内需的历史机遇，也面临结构性矛盾突出、资源环境约束加剧的诸多困难。总体上看，机遇多于挑战，有利条件多于不利因素，中原经济区建设上升为国家战略，必将使我们享受更多的优惠政策，增强商丘深化改革、扩大开放的整体活力，加快科学发展、可持续发展的进程。我们必须抓住机遇，克难攻坚，奋力为中原崛起、商丘振兴打基础、添活力、增后劲、创优势，不断开创经济社会发展的新局面。

今后我市经济社会发展的指导思想是：高举中国特色社会主义伟大旗帜，以邓小平理论和“三个代表”重要思想为指导，深入贯彻落实科学发展观，以科学发展为主题，以加快转变经济发展方式为主线，以富民强市为中心任务，按照建设中原经济区、加快中原崛起和河南振兴的总体战略部署，坚持“四个重在”的实践要领，扭住发展不放松，着力扩大内需，着力调整结构，着力改革开放，着力改善民生，实现经济持续较快发展和社会和谐稳定，为全面建成小康社会奠定坚实基础。

今后五年我市发展的蓝图和愿景是：综合实力明显增强。全市生产总值力争再翻一番，达到2300亿元，人均生产总值突破2万元，二、三产业增加值占生产总值比重达到80％以上，经济增长质量显著提高，综合实力在淮海经济协作区中的位次明显前移。三化协调格局明显优化。一批事关商丘未来发展的重大项目开工建设，新型工业化步伐加快。新型城镇化和新农村建设协调推进，全市城镇化率达到47％，接近全省平均水平。粮食生产核心区地位进一步巩固，综合生产能力提高到130亿斤左右。城乡居民收入明显增加。努力增加居民财产性收入，实现居民收入增长和经济发展同步、劳动报酬增长和劳动生产率提高同步，农民人均纯收入和城镇居民人均可支配收入增速超过全省平均水平，城乡居民日渐富裕，人民群众生活质量不断提高，满意度不断增加，幸福指数不断增强。和谐社会建设明显推进。教育、卫生、文化等基本公共服务体系不断完善，上学难、看病贵问题得到有效缓解，低收入群体住房条件明显改善，广覆盖、保基本、多层次、可持续的社会保障体系基本建立，民主法制和精神文明建设进一步加强，社会大局和谐稳定。我们相信，经过全市人民的共同努力，一个天蓝地绿水清宜居的生态之城，一个古代文化与现代文明交相辉映的魅力之城，一个创新创造、生机无限的活力之城，一个城乡协调发展、人民安居乐业的幸福之城，必将展现在人们面前，屹立在三商大地！

各位代表，《商丘市国民经济和社会发展第十二个五年规划纲要（草案）》及说明已印发各位代表，请一并审议。

我们坚信，有省委、省政府和市委的正确领导，有全市八百多万人民的齐心协力、奋勇拼搏，再大的困难也阻挡不了我们前进的步伐，我市“十二五”发展的宏伟蓝图一定能够变成美好的现实！商丘发展一定能够再创佳绩、再续辉煌！

三、2011年发展目标和重点工作

今年经济社会发展主要预期目标是：生产总值增速高于全省平均水平1到2个百分点，财政一般预算收入增速高于生产总值增速2个百分点，全社会固定资产投资增长20％，社会消费品零售总额增长17％，外贸出口增长21％，实际利用外商直接投资增长22％，居民消费价格涨幅控制在4％左右，人口自然增长率控制在6．5％。以内，新增城镇就业人员5．6万人，农民人均纯收入增长9％，城镇居民人均可支配收入增长9％，节能减排完成省定目标。

做好今年政府工作，要按照突出清醒与忧患，突出主题与主线，突出统筹与运作，突出民生与民心的总体要求，在大局中找准定位，抓住关键，讲求实效，努力实现“十个方面的突破”：抢抓机遇招商引资有新突破，推进大招商活动，成为承接产业转移的“桥头堡”；调结构促转型有新突破，加快产业和产品结构调整步伐，提高企业和产品的竞争力；推进产业集聚区建设有新突破，开展产业集聚区建设提升年活动，积极促进发展方式转变；综合交通体系建设有新突破，以建设交通枢纽为机遇，构建以高速公路、高速铁路和航空港为骨架的大交通格局；城中村改造有新突破，城乡面貌有新改观，城镇居民居住条件有新改善；新农村建设有新突破，改善农民群众生产条件和生活环境，统筹城乡协调发展；投融资机制建设有新突破，破解资金瓶颈制约；土地开发利用有新突破，保障发展用地需求；职业技能培训有新突破，提高劳动者就业创业技能，培育壮大人才队伍，增强承接产业转移的竞争力；社会保障水平有新突破，不断满足城乡居民日益增长的物质文化需求，让人民群众得到更多实惠。

实现上述目标任务，今年要着力抓好以下十个方面的工作：

（一）围绕保持经济较快发展，努力扩大需求。发挥投资、消费、出口对经济发展的协调拉动作用，加强经济运行调节，扩大需求规模，优化需求结构。加强重点项目建设。今年全市第一批拟安排重点建设项目243个，总投资1333亿元，当年计划投资354亿元。开展“项目突破年”活动，进一步完善重点项目建设五项机制，着力提高项目建设管理水平。精心谋划、筛选和编报项目，努力争取国家和省对我市的资金支持，加快推进中石油商丘千万吨炼油厂项目，加快在建重大项目建设进度，确保投资对经济发展的持续拉动作用。着力扩大消费需求。落实国家调整收入分配关系的各项政策，着力提高城乡低收入群众收入，扩大中等收入群体，增强居民消费能力。继续开展家电、摩托车下乡、家电以旧换新和万村千乡市场工程建设。培育楼宇经济、医疗保健、教育培训、家政服务以及信息、健身服务等消费热点。积极扩大出口，增加出口拉动经济发展的比重。加强经济运行调节，提高煤电油气运保障水平，统筹电力供需平衡，搞好成品油和天然气的稳定供应，确保居民生活、公共设施等重点领域需求。加强重点物资运输协调，做好重点时段的客货运输协调工作。加强市场物价管理，实施综合治理政策，加强对粮、油、肉、蛋、奶、蔬菜、液化气等生活资料和种子、化肥、柴油、农药等生产资料的市场价格监测，严厉打击囤积居奇和哄抬物价的行为，保持市场物价的基本稳定。继续强化企业服务，建立健全政策导向、市场开拓、融资服务、困难企业帮扶等长效机制，深入开展“企业服务年”活动，市、县分别抓好100家重点企业，帮助解决困难，开拓市场，提高效益。

（二）围绕促进经济更好发展，加快结构调整和转型升级。把调整优化产业产品结构作为紧迫任务，切实抓出成效，实现调结构促转型的新突破。提升传统产业，煤化工重点开发煤制乙二醇、聚甲醛等技术，拉长产业链，发展下游深加工产品；铝及铝的深加工围绕终端产品化，开发应用低温低电压铝电解技术、高端铝材生产技术，提高产品附加值；纺织服装加工业努力形成涵盖纺纱、织布、印染、面料、服装加工销售的产业链，打造中国有影响的纺织服装基地。发展优势产业，发挥粮食、果蔬、棉花和畜禽养殖等基础优势，以龙头企业为带动，大力发展以面品、肉奶和速冻食品加工为主的食品工业，力争规模以上食品工业完成增加值100亿元，打造国内有影响力的食品工业基地。制冷产业力争新入驻制冷相关企业10家以上，打造中国制冷产业基地。培育新兴产业，大力发展碳纤维、超硬材料、特种玻璃等新型功能材料，加快发展高性能纤维产品和复合材料、金刚石微粉及其制品等超硬材料产品、LOW－E浮法玻璃产品，形成一批科技含量高、市场前景好的新材料基地。力争今年金刚石产量6亿克拉、金刚石微粉8亿克拉；12K丝碳纤维产量200吨；优质浮法玻璃700万重量箱。壮大现代服务业，加快豫东综合物流集聚区建设，努力建设国家级战略储备中心。壮大现有金融机构，积极引进股份制银行，发展金融租赁公司、证券公司、保险公司等非银行金融机构。推进商丘古城的保护和开发，发展黄河故道生态旅游，培育县域旅游品牌，打造古色、绿色、红色、特色旅游精品景区。

（三）围绕拓宽发展空间，持续推进产业集聚区建设。按照“四集一转”的要求，把2011年作为产业集聚区建设提升年，完成投资330亿元以上，其中基础设施投资35亿元，工业项目投资260亿元；实现营业收入800亿元以上。提升基础设施承载功能，采取政府引导和社会化运作，积极推进产业集聚区投融资平台与金融机构的合作，扩大基础设施建设融资规模。全市每个产业集聚区基础设施年度完成投资3亿元以上。提升入驻项目数量质量，围绕特色主导产业，重点引进产业层次高、科技含量高的重大项目。认真执行投资强度、容积率、建筑密度等集约用地标准，严把规划、核准、土地、环保审批关，坚决杜绝违反国家产业政策、高污染和低水平、产能过剩项目进入。提升主导产业支撑能力，加快推进全市产业集聚区内136个工业项目建设，迅速做大制冷、食品、铝深加工、纺织服装、生物制药等特色主导产业规模，培育一批产业集群，力争主营业收入超100亿元的产业集聚区达到3个，超60亿元的产业集聚区达到5个。提升服务管理水平，加快推进污水处理厂、质量检测中心、电网、公租房等配套服务设施建设。提高投融资平台注册资本金，积极引进国内外产业基金、投资公司等战略投资者，扩大融资规模。完善集聚区管理机构，充实综合管理和公共服务职能，全面落实规划、项目审批、国土资源、环保、工商、统计等县级经济管理权限。

（四）围绕强化基础地位，大力发展现代农业。按照“强基础增效益，稳产量保供给，调结构促增收，建新村惠民生，抓改革添活力”的总体思路，积极做好“三农”工作。提高农产品供给能力，加快粮食核心区建设，认真实施国家新增1000亿斤粮食生产能力项目，确保全市粮食综合生产能力稳定在120亿斤以上。搞好粮食深加工，加快形成粮食加工集群效应，带动农民增收。推进新一轮“菜篮子”工程建设，确保农产品的有效供给，稳定市场物价，满足居民需要。提高农业产业化水平，积极培植农业产业化龙头企业，大力发展农民专业合作经济组织，改善农产品流通，不断提升农业发展的质量和效益。调整优化农业结构，大力发展果蔬、花卉等高效农业，加快标准化养殖小区建设，积极发展水产养殖业，提高优质、特色农畜产品比重。加快发展木材加工、家具、地板等林业产业，实现由资源型林业向产业型林业转变。推广先进适用技术，强化农业科技创新，加强农民培训，提高农民科技文化素质。抓好农田水利基本建设，完善引黄灌溉体系和井灌体系，加强灌区续建配套建设，实施节水改造工程，抓好郑阁、王安庄、马楼、任庄4座中型水库和刘口、洪河头2座小型水库除险加固工程建设项目，做好其他16座小型水库除险加固工程建设前期工作，力争新增库容8180万立方米，增强抗御自然灾害和旱涝保丰收的能力。落实强农惠农政策，财政用于农业农村的支出及时拨付到位，扩大补贴范围，提高补贴标准，加大农业政策性金融和保险对“三农”的支持力度。深化农村体制改革，继续推进农村综合改革和集体林权制度、县级供销社体制改革，建立健全区域性农技推广、动植物疫病防控、农产品质量监管等公共服务机构。

（五）围绕构建现代城镇体系，认真实施三年行动计划。按照“一核两翼组团发展，四位一体统筹推进”的城镇化发展战略，市本级集中力量抓好中心城区和商虞一体化建设，县一级集中力量抓好县城建设，结合重点乡镇和新型农村社区建设，统筹规划，分类指导，加快推进。着力抓好中心城区建设，今年是创建国家卫生城市的关键之年，以创建为载体，以内河治理、道路升级改造、公园建设为重点，完成城市道路、市容环卫、公共交通、供水、排水、燃气、集中供热、城市照明等8个专项规划。加快推进商虞一体化进程，以道路互联互通为重点，实现规划、功能一体化。全面启动城区生态水系规划建设，实施运河中段北岸沿河公共景观工程；做好主干道升级改造工程，对重点道路辅道实施罩面；投入2000万元以奖代补资金，继续实施背街小巷治理，解决好群众出行顺畅问题。推进月季公园、翠竹园等公园建设，巩固园林城创建成果，增加城市绿量。实施中水回用、垃圾渗沥液处置、污泥处置和粪便处理工程建设。加大旧城和城中村改造步伐，努力提升城市形象。开展城市精细化管理，深化环卫体制机制改革，探索建立多元化的环卫融资机制。加强市容环境卫生综合整治，逐步建立长效管理机制。全面提高市民健康文明素质，改善城乡面貌。着力抓好县城和中心镇区建设，结合区位、资源、人文、产业特点，科学定位县城发展，高起点、高标准地推进县城规划建设，实施联动互补组团发展。抓好道路、市场建设，做好污水处理和垃圾无害化处理，建设美丽宜居县域城区。中心镇区建设突出特色，加强与专业园区、新型社区建设的衔接，推进产城融合发展与农民就近就业转移，培育特色乡镇。鼓励因地制宜，放宽户籍限制，促进符合条件的外来务工人员进城落户，着力解决好他们的就业、安居、子女就学、社会保障等突出问题，逐步使进城落户农民真正变为市民，享有平等权益。着力抓好新型农村社区建设，继续落实好新农村建设、扶贫开发整村推进、农村土地综合整治和扶弱培强工程“四结合”工作机制，抓好103个新农村示范村和土地综合整治百村工程建设，提高基础设施和公共服务水平。产业集聚区内的村庄，率先实现城乡一体化，城市建成区内的城中村，全面实现城市化。着力抓好综合交通体系建设，争取尽快开工建设商丘中心火车站交通枢纽工程，扩大商丘货运编组规模，提高铁路运输能力。开工建设商丘机场和沱浍河工程。加快高速公路改扩建工程建设，加强国道升级改造，完善县乡公路设施，构建公路交通网络，促进区域间通道联网畅通。

（六）围绕增强发展活力，切实提高改革开放水平。推动改革开放向纵深发展，破解发展难题，增强发展动力和活力。加大改革攻坚力度，继续深化国有企业改革，完成市直部门所属企业的脱钩改制任务。鼓励支持民营经济发展，营造各种所有制经济依法平等使用生产要素、公平参与市场竞争、同等享受法律保护的体制环境。推进社会事业领域改革，继续深化医药卫生体制改革，实施教育改革发展规划纲要，建立事业单位岗位管理制度。完善水资源管理体制，加快水利工程建设和管理体制改革，继续推进水价改革。巩固扩大公安警务机制改革成果。破解瓶颈制约因素，坚持最严格的耕地保护制度。建立健全土地整治、节约集约用地和监督管理“三项机制”，大力开展农用地和村庄整治，稳妥推进城乡建设用地增减挂钩试点，探索农村宅基地有偿退出机制改革，加大存量建设用地盘活力度。坚持政府主导和市场化运作相结合，鼓励更多的社会资金进入担保领域，切实把投融资平台做好、做实、做强。着力扩大民间投资，采取政府购买服务、减免税费、以奖代补、贷款补贴等多种方式，引导民间资本进入基础设施、公用事业、保障性住房、社会事业等领域。落实人才培养使用规划，建立科学的人才评价机制、选拔任用机制、人才激励机制，完善人才创业扶持政策，营造良好的人才发展环境。持续开展大招商活动，深入研究产业转移规律，大力开展产业招商，打造富有特色的产业集群。紧盯央企、国企和大型民企，努力引进大项目、好项目。每个县（市、区）每年至少引进1个固定资产投资3亿元以上，且当年到位资金50％以上的工业项目。组织筹办第六届豫商大会，认真组织参加中部论坛、厦洽会、高交会等境内外招商活动。优化经济发展环境，严格落实优化发展环境责任制，坚决克服部门主义、本位主义，坚持凡是有利于优化发展环境的事情都要自觉执行、主动落实，不讲条件、不计得失；凡是涉及多个部门单位的事情，都要主动协调、密切配合，确保令行禁止、政令畅通。全面清理收费项目，规范对企业的检查行为，健全投诉受理机制，实现招商引资零障碍。

（七）围绕增强发展软实力，积极推进文化名市建设。大力弘扬“三商”文化，着力打造“华商之都”，为加快商丘发展提供文化支撑。积极发展文化产业，进一步提升商文化、火文化、根文化、汉文化等知名文化品牌。推进大运河（商丘段）和商丘古城世界遗产申报工作。重视文化经济，加快文化产业集聚区和文化园区建设，重点培育演艺娱乐、文物古玩、文化旅游、民间工艺美术等特色文化产品和优势文化产业，发展壮大商丘日报报业集团、商丘演艺集团、民权王公庄画虎村文化传播有限公司等骨干文化企业。大力发展文化事业，继续实施舞台艺术送农民活动、文化信息资源共享工程和农家书屋工程。加强文化基础设施建设，加快博物馆、体育中心、乡镇文化站、城市社区文化活动场所等项目建设进度，抓好县级文化馆、图书馆的新建、重建和翻新。推进文化体制改革，加快经营性文化事业单位改制步伐，鼓励民间资本参与改制重组。改革文化管理体制，推进文化管理部门和文化市场管理机构整合。加强重点文物、珍贵档案和非物质文化遗产的保护和管理，做好地方史志和社会科学研究工作。提高公民文明素质，深入推进社会公德、职业道德、家庭美德、个人品德建设，构建符合传统美德与时代要求的道德和行为规范。积极开展群众性精神文明创建活动，壮大志愿服务者队伍。净化社会文化环境，加强青少年思想道德建设。倡导爱岗敬业、诚实守信、勤俭自强、团结互助的社会风尚，引导人们知荣辱、讲正气、献爱心、树形象，形成良好的社会风气和价值导向。

（八）围绕增强可持续发展能力，扎实开展节能减排。正确处理节能减排与经济社会发展的关系，统筹协调，综合施治，确保完成节能减排任务。严格落实目标责任制，建立完善政府节能减排目标责任考评机制，建立健全淘汰落后产能的长效机制，有效降低企业的能源消耗和污染排放。加快循环经济试点建设，重点打造有色、煤炭、化工、农业和再生资源等领域循环产业链，认真落实循环经济财税、价格、投融资政策，创新循环经济发展模式。加快推进自主创新，积极发展创业中心、研发中心、孵化中心等创新载体，加快中科院新材料研发中心、电解铝节能研发中心建设进度。完善产学研合作机制和科技成果转化机制，加强知识产权保护，加速科技成果的转化应用。实施重大科技专项计划，开展科技攻关，解决我市主导产业发展的共性问题和关键技术，提升企业核心竞争力。加强环境保护，强化资源节约，促进资源综合利用，严格控制污染物排放总量，深化生态补偿机制。开展环境综合整治，完成饮用水水源地环境保护专项整治工作。按照“谁污染、谁负责”的原则，深化污染防治，促进清洁生产。抓好生态建设，大力开展植树造林，继续推进生态廊道网络、村镇绿化、农田防护林、城市防护林等林业生态工程建设，加强水资源保护和开发利用。

（九）围绕提高人民群众幸福指数，着力推进社会建设。发展社会事业，把教育摆在优先发展的战略地位，落实教育改革发展规划，加大教育经费投入，实施义务教育资源均衡配置工程、中小学校舍安全工程和学前教育三年发展规划。整合职业教育资源，建立示范性国家级高级技能培训基地和区域性公共实训基地。重视学科优化设置和教学科研质量提升，积极发展高等教育。加强公共卫生服务体系建设，提升城乡医疗服务能力和技术水平。广泛开展全民健身运动，发展竞技体育。统筹做好人口和计划生育工作，继续稳定低生育水平。推进全民创业，实施技能振兴计划，加强劳动者职业技能培训，提升劳动者就业能力，重点解决好城镇零就业家庭、农村贫困家庭和“4050”人员等困难群体的就业。完善社会保障体系，扩大城镇职工基本养老保险、基本医疗保险覆盖面，着力解决跨区域制度衔接问题，完善失业保险与城市低保制度的联动机制，提高城镇居民基本医疗保险、新型农村合作医疗、城乡低保和农村五保对象集中供养标准。积极开展新型农村社会养老保险试点工作，争取更多县（市、区）进入试点名单。大力发展慈善事业，建立完善覆盖城乡的社会救助体系。创新社会管理，推进平安商丘建设，开展社会治安综合治理，依法打击各类违法犯罪活动。搞好信访稳定工作，深入推进社会矛盾化解。落实安全生产责任制，坚决遏制重特大安全生产事故发生。加强食品药品监管，保障人民群众的健康安全。积极发展民族和宗教事业，加快少数民族地方经济发展。继续实施质量兴市战略，提升产品质量、工程质量、服务质量和环境质量。加强公共安全应急管理体系建设，增强公共应急水平，不断提高应对各种风险的能力。

（十）围绕解决关系城乡居民切身利益的问题，继续办好十件实事。1．缓解城区交通紧张状况。在火车南站和归德南路建设2处公交停车场，提高公交通行能力，方便市民出行；大修改造民主西路、平原北路、香君中路，新建睢阳北路、文化西路，改善路况路貌。2．优化城市居民居住环境。完成80条区管道路和背街小巷整修工程；加快旧城城中村改造速度，完成拆迁面积280万平方米，开工建设86万平方米；治理早期人防工程，改建面积1200平方米；把包河、运河水变清，启动实施对忠民河、康林河综合治理；建成应天公园；新建固定垃圾中转站50个，水冲公厕100个，沿街单位厕所对市民开放，解决群众如厕难问题。3．改善农村生产生活条件。修建农村公路300公里，架设骨干河道和干支沟桥梁80座；建设户用沼气池1万座；解决50万人安全饮水问题；重点扶持103个新农村试点村建设；启动新一轮农村电网改造升级工程，保障农村生产、生活用电。4．提高低收入群体住房保障能力。建设廉租房5000套、经济适用房3000套，提供公共租赁房3000套，实施廉租补贴2万户。5．加强职业技能和就业培训。实施“阳光工程”和“雨露计划”，完成各类职业技能和就业培训14万人；政府购买公益性岗位5000个，帮助“4050”人员再就业。6．完善城市公共服务功能。开工建设城市规划展览馆，筹备建设科技文化艺术中心、老年活动中心、青少年宫。7．增强医疗卫生服务能力。开工建设市卫生监督所，新建第二人民医院病房楼和门诊楼项目。8．加强中小学校建设和幼儿教育。改造回民中学D级危房4800平方米，架设连接南北校区的过街天桥；在睢阳区和开发区城区内分别启动一所中小学校建设；新建市实验幼儿园教学综合楼；更换市直中小学黑板、桌凳；新建市职教中心学生餐厅。9．加大弱势群体救助力度。实施“贫困白内障患者复明工程”，开展复明手术3000人；对城市无经济来源、无劳动能力、无赡养人的老人进行救助，对大病特困儿童进行救助，对孤儿、重度残疾儿童每月定额发放生活补贴。10．提高社会保障水平。企业退休人员基本养老金在2010年基础上，人均月增加190元；新型农村合作医疗和城镇居民基本医疗保险财政补贴标准，由人均120元提高到200元；城市低保对象补差标准由每人每月145元提高到不低于160元，农村低保对象由60元提高到不低于70元；五保集中供养标准由每人每年2000元提高到2240元。

四、切实加强政府自身建设

坚持“三具两基一抓手”，深入开展“两转两提”和“创先争优”活动，不断提高政府工作人员的能力素质，建设人民满意政府。

（一）转变政府职能。立足商丘实际，注重调查研究，遵循客观规律，着眼长远发展，努力提高决策的科学化、民主化，力争少走弯路，不走错路。大力开展行政效能监察，推行行政审批事项限时办结制，反对推诿扯皮，追究失职渎职行为，提高工作效率。进一步精简审批事项，减少审批环节，缩短审批时间，建立分工明确、运转协调、行为规范的管理体系和工作机制。

（二）推进依法行政。严格按照法定权限和程序行使权力、履行职责。认真执行人大及其常委会的决议、决定，自觉接受人大法律监督、政协民主监督和人民群众的社会监督，认真办理人大代表建议、议案和政协委员提案。遵循政府工作规则，完善重大事项集体决策、专家咨询制度。严格行政执法责任制度和过错追究制度，强化行政执法监督，促进公正执法、文明执法。全面推行和完善政务公开、事务公开，确保权力在阳光下运行。

（三）改进工作作风。坚持说实话、办实事、求实效，反对弄虚作假，做表面文章。深入实际、深入群众，办好事关经济社会发展的大事、关系群众切身利益的实事、影响社会和谐的要事。精简文件、会议，减少不必要的应酬，集中精力抓落实，凝神聚力促发展，埋头苦干求实效。

（四）坚持从严治政。加强公务员队伍教育管理，提高素质能力，增强服务意识。加强财政管理，严格控制一般性支出，坚持厉行节约，反对铺张浪费，把有限的资金用在推动事业发展上，用在保障改善民生上。严厉查处不作为、乱作为和违法违纪行为，坚决纠正损害群众利益的不正之风，真心实意为群众排忧解难，维护好广大人民的根本利益。

各位代表，“十一五”的成绩令人鼓舞，“十二五”的蓝图催人奋进。在全面建设中原经济区的大潮中，我们一定牢记使命，履职尽责，锐意进取，踏实工作，为开创“十二五”发展新局面，为建设更加美好的新商丘而努力奋斗！
